# Supplementary material for: Diel transcriptional responses of coral-Symbiodiniaceae holobiont to elevated temperature
Source: Commun Biol. 2024 Jul 19;7:882. doi: 10.1038/s42003-024-06542-6 (PMC11271600; doi:10.1038/s42003-024-06542-6)
Supplement: Supplementary file 2 — Supplementary Information [file 42003_2024_6542_MOESM2_ESM.pdf]

Diel transcriptional responses of coral-Symbiodiniaceae holobiont to elevated temperature

Sanqiang Gong[1,2], Jiayuan Liang[2], Lijia Xu[4], Yongzhi Wang[4], Jun Li[1], Xuejie Jin[1], Kefu Yu[2,3*], Yuehuan Zhang[1*]

[1]Key Laboratory of Tropical Marine Bio-resources and Ecology & Guangdong Provincial Key Laboratory of Applied Marine Biology, South China Sea Institute of Oceanology, Chinese Academy of Sciences, Guangzhou 510301, China

[2]Guangxi Laboratory on the Study of Coral Reefs in the South China Sea, Coral Reef Research Center of China, School of Marine Sciences, Guangxi University, Nanning 530004, China

[3]Southern Marine Science and Engineering Guangdong Laboratory (Guangzhou), Guangzhou 511458, China

[4]South China Institute of Environmental Sciences, The Ministry of Ecology and Environment of PRC, Guangzhou, 510530, China

*****Corresponding author:

Kefu Yu

Email: kefuyu@scsio.ac.cn

Yuehuan Zhang

Email: yhzhang@scsio.ac.cn

Figure S1. Diel oscillation patterns of candidate circadian clock-controlled genes in the *A. tenuis-Cladocopium* sp. holoboint. Oscillation plots for core rhythmic genes cycling with a 24-hour period in host (A) and symbiont (B) under DD, LD and HLD conditions.

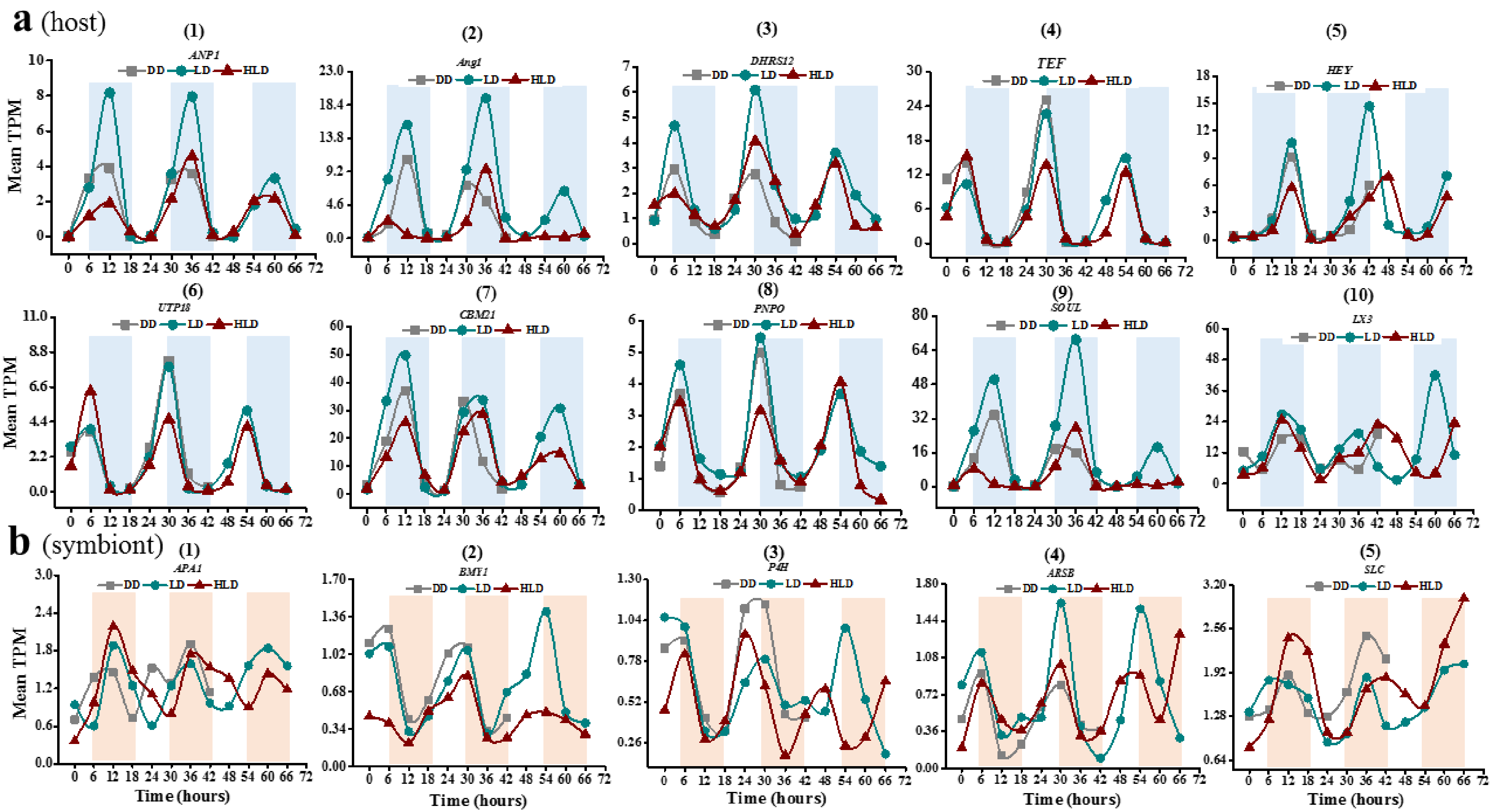
a (host)
(1) ANP1
(2) Ang1
(3) DHRS12
(4) TEF
(5) HEY
(6) UTP18
(7) CBM21
(8) PNPO
(9) SOUL
(10) LX3
b (symbiont)
(1) APA1
(2) BMY1
(3) P4H
(4) ARSB
(5) SLC
DD
LD
HLD
Mean TPM
Time (hours)
