# Supplementary material for: Diel transcriptional responses of coral-Symbiodiniaceae holobiont to elevated temperature
Source: Commun Biol. 2024 Jul 19;7:882. doi: 10.1038/s42003-024-06542-6 (PMC11271600; doi:10.1038/s42003-024-06542-6)
Supplement: Supplementary file 3 — Description of Additional Supplementary Files [file 42003_2024_6542_MOESM3_ESM.pdf]

Description of Additional Supplementary Files

File name: Supplementary Data 1
Description: Rhythmicity test of algal density, photosynthetic efficiency, reactive oxygen species (ROS) leakage, and lipid peroxidation in the A. tenuis holobiont (Cosinor algorithm) under DD, LD and HLD conditions.

File name: Supplementary Data 2
Description: Gene list of identify rhythmic genes in coral host.

File name: Supplementary Data 3
Description: Gene list of identify rhythmic genes in symbiont.

File name: Supplementary Data 4
Description: List of clock genes (C) or clock-controlled genes (CCG)identified in coral and symbiont.
