# Supplementary material for: Diel transcriptional responses of coral-Symbiodiniaceae holobiont to elevated temperature
Source: Commun Biol. 2024 Jul 19;7:882. doi: 10.1038/s42003-024-06542-6 (PMC11271600; doi:10.1038/s42003-024-06542-6)
Supplement: Supplementary file 8 — Reporting Summary [file 42003_2024_6542_MOESM8_ESM.pdf]

nature portfolio

Corresponding author(s): Sanqiang Gong

Last updated by author(s): Apr 1, 2024

Reporting Summary

Nature Portfolio wishes to improve the reproducibility of the work that we publish. This form provides structure for consistency and transparency in reporting. For further information on Nature Portfolio policies, see our Editorial Policies and the Editorial Policy Checklist.

Statistics

For all statistical analyses, confirm that the following items are present in the figure legend, table legend, main text, or Methods section.

n/a	Confirmed	
☒	☐	The exact sample size (*n*) for each experimental group/condition, given as a discrete number and unit of measurement
☐	☒	A statement on whether measurements were taken from distinct samples or whether the same sample was measured repeatedly
☐	☒	The statistical test(s) used AND whether they are one- or two-sided<br>*Only common tests should be described solely by name; describe more complex techniques in the Methods section.*
☐	☒	A description of all covariates tested
☐	☒	A description of any assumptions or corrections, such as tests of normality and adjustment for multiple comparisons
☐	☒	A full description of the statistical parameters including central tendency (e.g. means) or other basic estimates (e.g. regression coefficient) AND variation (e.g. standard deviation) or associated estimates of uncertainty (e.g. confidence intervals)
☐	☒	For null hypothesis testing, the test statistic (e.g. *F*, *t*, *r*) with confidence intervals, effect sizes, degrees of freedom and *P* value noted<br>*Give P values as exact values whenever suitable.*
☒	☐	For Bayesian analysis, information on the choice of priors and Markov chain Monte Carlo settings
☒	☐	For hierarchical and complex designs, identification of the appropriate level for tests and full reporting of outcomes
☒	☐	Estimates of effect sizes (e.g. Cohen's *d*, Pearson's *r*), indicating how they were calculated

Our web collection on statistics for biologists contains articles on many of the points above.

Software and code

Policy information about availability of computer code

Data collection	1. The reef-building coral A. tenuis (measuring 10 to 20 cm) were collected from a depth of 3- to 4-m on a tropical coral reef in Sanya (109° 29’ E, 18° 12’ N), Hainan Island, China.<br>2. the data sets of Symbiodiniaceae cell density and maximal PSII quantum yield (FV/FM) were monitored using a hemocytometer counts under a microscope (CX21, Olympus, Japan) and a Diving-PAM-II<br>3. The fluorescence intensity of CellROX was quantified using a SpectraMax to calculate the relative release of ROS by coral symbiont.<br>4. The level of oxidation stress in the tissues of the coral host were quantified by monitor the total malondialdehyde content.<br>5. the data of RNA-seq was generated by NovaSeq 6000
Data analysis	SqueezeMeta software, an automatic pipeline for metagenomics/metatranscriptomics analysis, was used for analyzing NGS-data sets.

For manuscripts utilizing custom algorithms or software that are central to the research but not yet described in published literature, software must be made available to editors and reviewers. We strongly encourage code deposition in a community repository (e.g. GitHub). See the Nature Portfolio guidelines for submitting code & software for further information.

Data

Policy information about availability of data

All manuscripts must include a data availability statement. This statement should provide the following information, where applicable:

- Accession codes, unique identifiers, or web links for publicly available datasets
- A description of any restrictions on data availability
- For clinical datasets or third party data, please ensure that the statement adheres to our policy

The raw sequence data of RNA sequencing libraries generated during the current study were deposited in the Sequence Read Archive (PRJNA1026720) of the NCBI (https://blast.ncbi.nlm.nih.gov). ALL data that support the findings of this study are openly available

Human research participants

Policy information about studies involving human research participants and Sex and Gender in Research.

Reporting on sex and gender	no human participants
Population characteristics	no human participants
Recruitment	no human participants
Ethics oversight	no human participants

Note that full information on the approval of the study protocol must also be provided in the manuscript.

Field-specific reporting

Please select the one below that is the best fit for your research. If you are not sure, read the appropriate sections before making your selection.

☐ Life sciences ☐ Behavioural & social sciences ☒ Ecological, evolutionary & environmental sciences

For a reference copy of the document with all sections, see nature.com/documents/nr-reporting-summary-flat.pdf

Ecological, evolutionary & environmental sciences study design

All studies must disclose on these points even when the disclosure is negative.

Study description	Diel transcriptional response of coral-Symbiodiniaceae holobiont to elevated temperature
Research sample	Two groups: one group under normal temperature, the other under elevated temperature
Sampling strategy	Random Sampling was used to sample coral (n=20) colonies, which were fragmented into branches (measuring height ~5 cm, total of ~212 branches). these branches were randomly grouped.
Data collection	1. the data sets of Symbiodiniaceae cell density and maximal PSII quantum yield (FV/FM) were monitored using a hemocytometer counts under a microscope (CX21, Olympus, Japan) and a Diving-PAM-II<br>2. the data of RNA-seq was generated by NovaSeq 6000
Timing and spatial scale	Data sets were collected during November 6 to 15 of 2021.
Data exclusions	no
Reproducibility	At least three replicates were designated in each time point to validate significance differences and reproducibility
Randomization	212 branches of coral were randomly allocated into two different groups.
Blinding	no

Did the study involve field work? ☐ Yes ☒ No

Reporting for specific materials, systems and methods

We require information from authors about some types of materials, experimental systems and methods used in many studies. Here, indicate whether each material, system or method listed is relevant to your study. If you are not sure if a list item applies to your research, read the appropriate section before selecting a response.

Materials & experimental systems

n/a	Involved in the study
☒	☐ Antibodies
☒	☐ Eukaryotic cell lines
☒	☐ Palaeontology and archaeology
☒	☐ Animals and other organisms
☒	☐ Clinical data
☒	☐ Dual use research of concern

Methods

n/a	Involved in the study
☒	☐ ChIP-seq
☒	☐ Flow cytometry
☒	☐ MRI-based neuroimaging
